# Dietary phytochemical index associated with cardiovascular risk factor in patients with type 1 diabetes mellitus

**DOI:** 10.1186/s12872-021-02106-2

**Published:** 2021-06-12

**Authors:** Saeideh Delshad Aghdam, Fereydoun Siassi, Ensieh Nasli Esfahani, Mostafa Qorbani, Asadollah Rajab, Zahra Sajjadpour, Anahita Bashiri, Maryam Aghayan, Gity Sotoudeh

**Affiliations:** 1 Department of Community Nutrition, School of Nutritional Sciences and Dietetics, Tehran University of Medical Sciences, Hojatdost Street, Naderi Street, Keshavarz Blv, Tehran, 14167-53955 Iran; 2 Diabetes Research Center, Endocrinology and Metabolism Clinical Sciences Institute, Tehran University of Medical Sciences, Tehran, Iran; 3 Non-communicable Diseases Research Center, Alborz University of Medical Sciences, Karaj, Iran; 4 Chronic Diseases Research Center, Endocrinology and Metabolism Population Sciences Institute, Tehran University of Medical Sciences, Tehran, Iran; 5 Iranian Diabetes Association, Tehran, Iran; 6 Nutrition and Endocrine Research Center, Research Institute for Endocrine Sciences, Shahid Beheshti University of Medical Sciences, Tehran, Iran

**Keywords:** Diabetes mellitus, cardiovascular diseases, phytochemicals, adult, hyperglycemia

## Abstract

**Background:**

Dietary phytochemical index (DPI) is useful and inexpensive method to identify the role of phytochemicals on cardiovascular disease (CVD) risk factors. This study aimed to assess the relationship between DPI and CVD risk factors in patients with type1 diabetes mellitus.

**Methods:**

A total of 261 participants aged 18–35 years with T1DM were enrolled in this cross-sectional study to assess the relationship between DPI and CVD risk factors. Anthropometric measurements, blood lipids, glucose, and antioxidant level were measured. Food intakes were determined using a food frequency questionnaire to calculate DPI. Logistic regression was used.

**Results:**

The mean age of participants was 25 years. After adjustment for potential confounders, participants in the highest tertile of DPI had 88 % lower chance of hyperglycemia (*P* for trend = 0.020), 81 % lower chance of low high-density lipoprotein cholesterol (HDL-C) (*P* for trend = 0.030) and 98 % lower chance of high low-density lipoprotein cholesterol to HDL-C ratio (*P* for tend = 0.040). There were no relationships between DPI and other CVD risk factors.

**Conclusions:**

Although higher intake of phytochemical-rich foods had a beneficial effect on some risk factors of CVD, more studies more studies are warranted to corroborate the present findings.

## Introduction

Diabetes mellitus (DM) is one of the most important cause of death worldwide, especially in developed countries. It is associated with the risk of hypertension (HTN), hypercholesterolemia, and cardiovascular diseases (CVD) [1]. Seven to 12% of patients who suffer from diabetes have type 1 DM (T1DM) [2]. Genetic and environmental triggers including toxins, viral infections, and some dietary factors may affect incidence of T1DM. Unfortunately, there are currently no preventive measures for the prevention of T1DM, so the reduction of its complications must be focused [2].

Oxidative stress (OS) as an imbalance between oxidants and anti-oxidants in the favor of the oxidants, leading to a disruption of redox signaling and control and/or molecular damage. OS plays an important role in the major diabetic complications, including retinopathy, nephropathy, neuropathy and accelerated coronary artery disease [3]. Insulin deficiency, in turn leads to chronic hyperglycemia and an increase level of lipid and protein peroxidation products, oxidative DNA damage markers, and lower activity of antioxidant enzymes, subsequently [4]. Some evidence showed that antioxidant capacity impairs in T1DM due to lower consumption of antioxidant components or lower levels of antioxidant substances [5].

Phytochemicals are mainly found in plant foods such as fruits, vegetables, cereals, soy and tea [6], which is inversely associated with chronic diseases including DM, CVD, and some cancers [7]. The beneficial effects of phytochemicals can be attributed to their antioxidant and anti-inflammatory properties, their effect on cell cycle regulation, hormones, vascular endothelium and immune cells [8]. Dietary phytochemical index (DPI) is a quantitative index defined as percentage of energy intake that are derived from phytochemicals rich foods in order to identify the role of phytochemicals on health [9].

Previous studies investigated the association of DPI with chronic diseases risk factors. Most of these studies conducted on healthy individuals, except of 2 case-control studies on women with breast cancer [10] and individuals with pre-diabetes [11]. The result of these studies showed inverse association between DPI and waist circumference (WC), body mass index (BMI), HTN, glucose intolerance, chance of pre-diabetes, dyslipidemia, and blood biomarkers of OS [11–13].

Few studies investigated the relationship between DPI and health outcomes and none of them were performed in patients with T1DM, who have complex conditions such as impaired glucose and lipid disturbances [14]. We hypothesis that DPI may have a beneficial relationship with the CVD risks; therefore, the aim of this study was to determine the relationship between DPI with CVD risk factors including blood glucose, lipid profile, antioxidant levels, blood pressure and anthropometric measurements in patients with T1DM.

## Materials and methods

### Study design

The present study is part of a cross-sectional study, which investigated the association between dietary patterns with CVD risk factors and OS in patients with T1DM with the approval of Ethics Committee of Tehran University of Medical Sciences (IR.TUMS.REC.1394.1595), and written informed consent was obtained from all the participants. The study was conducted on 273 patients with T1DM from the Iranian diabetes society and Endocrine and Metabolism Research Institute of Tehran University of Medical Sciences in Tehran, Iran. According to the exclusion criteria (individuals who had reported energy intake outside the range of 500–3500 kcal/day in women and 800–4200 kcal/day in men) 261 patients were finally analyzed for the non-biochemical assessments and due to financial limitation, 81 patients recruited for biochemical examination using simple random sampling method. Inclusion criteria were patients who were diagnosed with T1DM for at least six months, aged between 18 and 35 and hemoglobin A1c (HbA1c) ≤ 8%. Participants were excluded if they had BMI ≥ 40 kg/m^2^, drugs intake other than insulin to lower blood glucose, diagnosed CVD, cancer, kidney or liver disease, use of contraceptives, hormones and recombinant drugs, thyroid control drugs, taking weight reducing agents, anti-depressants and anti-anxiety drugs, use of any smoking (cigarette, hookah, tobacco pipe) and pregnancy or lactation.

### Demographic and general characteristics

Information about age, sex, duration of DM, educational level, daily insulin dose, type of insulin and dietary supplements intake were collected by questionnaires.

### Dietary intake assessment

Individual’s typical dietary intake, during the last year, was evaluated using a semi-quantitative food frequency questionnaire (FFQ) with 147 food items, interviewed by a trained dietitian. The validity and reproducibility of the FFQ were determined previously for fruits, vegetables, and energy intake [15]. In this FFQ, there is a list of food items with a standard serving size that Iranian people commonly consume [16]. Individuals were asked to report their usual frequency and amount of consumption of food items listed by day, week, and month over the previous year. The values listed for each food item were converted to gram using US Department of Agriculture (USDA) serving sizes whenever possible; if this was not possible, household measures were chosen and were then converted to grams. Energy and nutrient contents of food items were obtained from USDA food composition tables (FCTs) because Iranian FCTs are incomplete. The Iranian FCT was used for traditional food items that are not listed in the USDA FCT. Analyzing the energy and nutrients of each food item was done with the Nutritionist IV software version 3.5.1, that modified for Iranian food [17].

DPI is calculated based on the division of energy content of foods rich in phytochemicals on the total daily energy intake × 100, based on McCarty formula ($DPI = \frac{Dietary~energy~derived~from~phytochemical-rich~foods~{\text{(kcal)}}}{Total~daily~energy~intake {\text{(kcal)}}} \times 100$) [9]. To calculate the DPI, all the phytochemical-rich foods including fruits, vegetables (except potatoes), whole grains, legumes, fruit and vegetable juice, soy products, nuts, seeds, olive and olive oil were gathered from the FFQ [13].

### Clinical assessment

The weight was measured by a nutritionist using a digital scale (GAIA 359 PLUS. Jawon Medical Co. Ltd., Gyeongsan, Korea), to the nearest 100 g, with minimal dress and no shoes. Height was measured to the nearest 0.5 cm with a tape while the patient standing without shoes. The BMI was calculated by dividing the weight (kg) by height (square meter). WC was measured using an elastic tape measuring midpoint between the iliac crest and lowest rib. Blood pressure was measured from the right hand of the participants to the nearest 2 mmHg, after at least 10 min rest, while sitting on a chair with a mercury sphygmomanometer. Overweight and obesity defined as BMI = 25–29.9 kg/m^2^ and BMI ≥ 30 kg/m^2^, respectively, central obesity as WC ≥ 80 cm in women and ≥ 94 cm in men. HTN was defined as blood pressure ≥ 140/90 mmHg [18].

### Physical activity assessment

Physical activity of participants, during the previous week, was measured using the short form of International Physical Activity Questionnaire (IPAQ) [19]. The average time that a person would normally spend on different activities each day was asked. Then, to measure the value of metabolic equivalent task (MET)-hour/week, the sum of frequency and duration of activities multiplied by the MET of activity. The reliability and validity of IPAQ was assessed previously [19].

### Laboratory measurements

After a 12–14 h fasting at night, a trained nurse collected 5 mL of venous blood from the participants. Blood samples were collected in 2 separate tubes. One of the tubes was for separating serum and another tube containing ethylenediaminetetraacetic acid was used to separate the plasma. In order to separate the plasma samples from cells, the blood was centrifuged in 3000 rounds for 10 min. Then the remaining blood was washed three times with sodium chloride solution 0.9 g/L. Separation of cell membranes was performed by centrifugation for 5 min at 4 °C. Then hemolytic cells were used to determine the activity of antioxidant enzymes. The serum was separated from the blood by centrifugation for 10 min at 4 °C. After that, all the blood samples were stored at − 79 °C. Blood glucose measurements were performed on the day of the test. This study was conducted with observance of the Declaration of Helsinki and the National Ethical Guidelines in Biomedical Research in Iran.

Measurements of serum glucose and lipids were performed, using Pars Azmoon kit (Pars Azmoon, Tehran, Iran). The measurement of triglyceride (TG) levels was conducted by colorimetric and photometry. Fasting blood glucose (FBG) and total cholesterol (TC) measurement were conducted by enzymatic colorimetric and single point with photometric method. The measurement of total antioxidant capacity (TAC) and activity of glutathione peroxidase (GPx) and superoxide dismutase (SOD) were measured by commercial kits following the manufacturer’s protocol (ZellBio GmbH, Lonsee, Germany). The intra- and inter-assay coefficients of variation for SOD, GPx, and TAC were 5.8 and 7.2%, 3.5 and 4.7%, and 3.4 and 4.2%, respectively. The following formula was used for calculating low-density lipoprotein cholesterol (LDL-C) [37]: LDL-C = TC − high-density lipoprotein cholesterol (HDL-C) − TG/5.0 (mg/dL).

Hypertriglyceridemia was defined as serum TG ≥ 150 mg/dL (1.7 mmol/L), low HDL-C as serum HDL-C < 40 mg/dL (1.0 mmol/L) for men and < 50 mg/dL (1.3 mmol/L) for women, high LDL-C as serum LDL-C ≥ 100 (2.6 mg/dL), hypercholesterolemia as TC > 200 mg/dL, high HbA1c as HbA1c ≥ 7, hyperglycemia as FBG ≥ 100 mg/dL and high LDL-C/HDL-C ratio as LDL-C/HDL-C > 3 in women and > 3.5 in men [18, 20, 21].

### Statistical analysis

Statistical analysis was performed using SPSS software for Windows (version 23; SPSS Inc., Chicago, IL, USA; 2015). Individuals who had reported energy intake outside the range of 500–3500 kcal/day in women and 800–4,200 kcal/day in men were excluded from the study [22]. In this order 3 men and 9 women were excluded from the data analysis and the final analysis was done on 261 participants. At first, energy‑adjusted DPI was determined by residual method, then according to DPI, participants were categorized into tertiles. Normality of data distribution was tested using graph and Kolmogorov–Smirnov test. Comparison of the general and nutritional characteristics of the participants between the tertiles of energy‑adjusted DPI was done using analysis of variance (ANOVA) or χ^2^ test depending on the type of variables. Means ± SD of CVD risk factors across tertiles of DPI were compared by ANOVA test for crude model and analysis of covariance for adjusted models which adjusted for age, sex, total energy intake (kcal/day), physical activity (MET/hour/week), BMI (kg/m^2^), diabetes duration (year), total daily insulin dose, education and dietary supplement intake. Further adjustment for intake of saturated fatty acids (SFA), mono-unsaturated fatty acids (MUFA), poly-unsaturated fatty acids (PUFA) and trans fatty acids was done for lipids levels. In addition, dietary intake of sodium and potassium was adjusted for systolic blood pressure (SBP) and diastolic blood pressure (DBP). Logistic regression test was used in crude and adjusted model to determine the odds ratio (OR) of cardiovascular risk factors and their 95% confidence interval (CI) in each tertile of DPI, which adjusted for age, sex, total energy intake (kcal/day), physical activity (MET/hour/week), BMI (kg/m^2^), diabetes duration (year), total insulin dose (unit/day), education, dietary supplement intake. In all analyzes, the first tertile of DPI was considered as a reference and OR of the cardiovascular risk factor in the other tertiles was calculated towards it. Furthermore, to determine the overall trends of OR across increasing tertiles of DPI, the median of each tertile was used instead of the number of tertiles. *P *value lower than 0.05 was considered significant.

## Results

### Characteristics of participants across tertiles of DPI

**Fig. 1:**
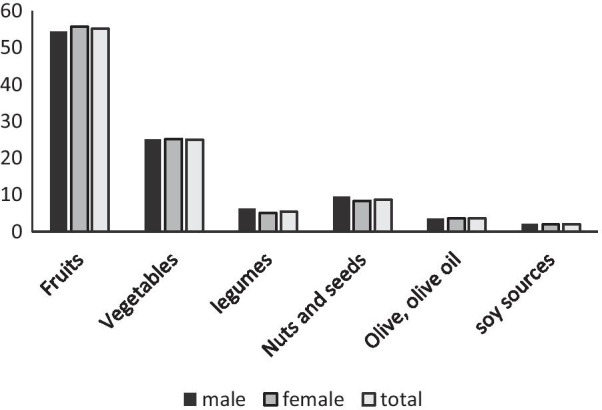
Contributions of different phytochemical‑rich food groups to energy‑adjusted dietary phytochemical index

The mean ± SD age of participants was 25 ± 5.4 years and 62.1% of the participants were women. DPI in the first, second and third tertiles of energy‑adjusted DPI was < 31.75, 31.75–40.06, and > 40.06, respectively for females and < 27.73, 27.73–36.05, and > 36.05, respectively for males. The characteristics of participants across tertiles of DPI are shown in Table 1. Participants in the lower tertile of DPI were significantly younger (*P *trend = 0.02). Other characteristics including gender, education, short-acting insulin intake, long-acting insulin intake, dietary supplement intake, daily insulin dose, BMI, weight, WC, diabetes duration, energy intake and physical activity were not significantly different across DPI tertiles (*P * trend > 0.05). The relative contributions of different phytochemicalrich food groups of DPI are shown in Fig. 1. The percentage of daily intake of energy from fruits and vegetables were higher than the other sources of phytochemical rich foods.

**Table 1:** Characteristics of participants across tertiles of the sex-specific energy‑adjusted DPI in patients with type 1 diabetes

Variables	DPI tertiles				*P *trend
	T1 (n = 87)	T2 (n = 87)	T3 (n = 87)	Total (n = 261)	
Gender					
Men	33 (33.3)	33 (33.3)	33 (33.3)	99	1.000^a^
Women	54 (33.3)	54 (33.3)	54 (33.3)	162	
Age (yrs)	24.3 ± 5.1	24.7 ± 5.4	26.0 ± 5.4	25.0 ± 5.4	0.022^b^
Weight (kg)	66.3 ± 12.3	65.9 ± 11.8	65.5 ± 11.6	65.9 ± 11.8	0.638^b^
Waist circumference (cm)	86.0 ± 10.2	84.8 ± 10.3	85.7 ± 9.8	85.5 ± 10.1	0.828^b^
BMI (kg/m²)	23.4 ± 3.2	23.5 ± 3.5	23.5 ± 3.1	23.4 ± 3.3	0.857^b^
Diabetes duration (yrs)	11.2 ± 6.0	12.6 ± 6.6	12.6 ± 6.7	12.1 ± 6.4	0.168^b^
Insulin dose (unit/day)	48.5 ± 15.4	48.8 ± 16.7	47.0 ± 20.5	48.1 ± 17.6	0.590^b^
Short-acting insulin intake					
Aspart	18 (34.3)	20 (32.8)	19 (32.8)	57	0.908^a^
Regular/mix	69 (31.6)	66 (35.1)	66 (33.3)	201	
Long-acting insulin intake					
Glargine/Detemir	18 (31.0)	21 (36.2)	19 (32.8)	58	0.814^a^
NPH/mix	68 (34.8)	63 (32.4)	66 (32.8)	197	
Dietary supplement intake					
Yes	31 (34.8)	32 (36.0)	26 (29.2)	89	0.564^a^
No	56 (32.7)	54 (31.6)	61 (35.7)	171	
Education					
Diploma and lower	27 (39.7)	22 (32.4)	19 (27.9)	68	0.382^a^
Academic	60(31.3)	64 (33.3)	68 (35.4)	192	
Physical activity (MET-hour/week)	26.2 ± 27.5	23.8 ± 26.5	27.3 ± 31.2	25.8 ± 28.4	0.804^b^
Energy intake (kcal/day)	2287.1 ± 632.9	2476.6 ± 680.3	2368.7 ± 732.4	2377.5 ± 684.9	0.433^b^
Dietary phytochemical index	23.6 ± 5.0	34.3 ± 3.2	45.8 ± 6.6	34.6 ± 10.4	< 0.001

Data are shown as the mean ± SD or number (%)

*DPI* dietary phytochemical index, *BMI* body mass index, *NPH* neutral protamine Hagedorn, no description, *MET* metabolic equivalent

^a^*P *value is for χ^2^ test, used for categorical data

^b^*P *trend is for analysis of variance test, used to compare continuous variables, calculated by linear regression test

### CVD risk factors across tertiles of DPI

**Fig. 2:**
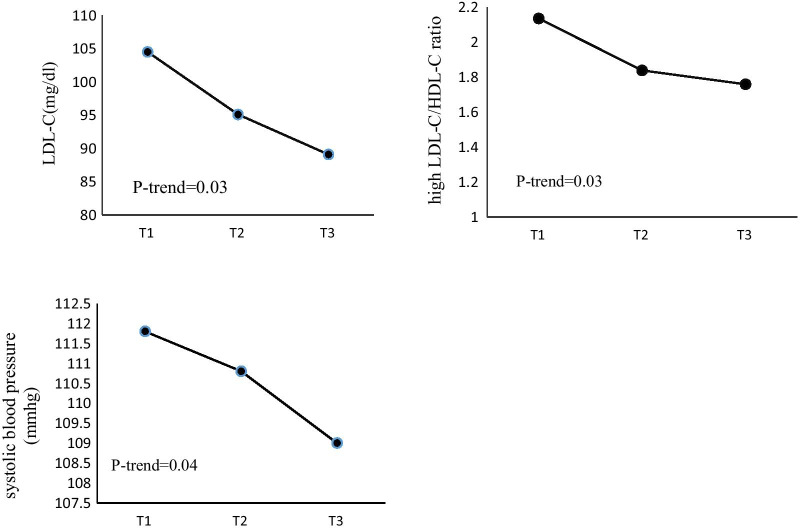
Multivariate-adjusted means of cardiovascular risk factors across tertiles of dietary phytochemical index determined by general linear model with adjustment for age, sex, total energy intake (kcal/day), physical activity (metabolic equivalent task/hour/week), body mass index (kg/m^2^), diabetes duration (year), insulin dose (unit/day), education and dietary supplement intake. LDL-C and LDL-C/HDL-C were further adjusted for intake of saturated fatty acids, mono-unsaturated fatty acids, poly-unsaturated fatty acids, and trans fatty acid. SBP further adjusted for dietary intake of sodium (mg) and potassium (mg). *LDL-C* low-density lipoprotein cholesterol, *HDL-C* high-density lipoprotein cholesterol, *SBP* systolic blood pressure

Table 2 presents the mean ± SD of CVD risk factors across tertiles of DPI. In the crude model, there was not any significant association between CVD risk factors and DPI (*P *trend > 0.05) (Model 1). After adjustment for age, sex, total energy intake, physical activity, BMI, diabetes duration, insulin dose, education and dietary supplement intake, participants in the higher tertile of DPI had significantly lower LDL-C to HDL-C ratio compared with those in the lower tertile (*P *trend = 0.03) (Model 2). In addition, after further adjustment for intake of SFA, MUFA, PUFA and trans fatty acids participants in the higher tertile of DPI, had significantly lower LDL-C (*P *trend = 0.03) and LDL-C to HDL-c ratio (*P *trend = 0.03) compared with those in the lower tertile (Model 3). After further adjustment for dietary intake of sodium and potassium, significant relation was observed between DPI and SBP (*P *trend = 0.04) (Model 3) (Fig. 2). No significant association was found between GPx, SOD, TAC and DPI. By increasing the tertiles of DPI, levels of FBG decreased, while TAC increased, however these findings were not statistically significant (*P *trend > 0.05).

**Table 2:** Cardiovascular risk factors across tertiles of the sex-specific energy-adjusted DPI in patients with type 1 diabetes (n = 81)

Variable	DPI tertiles			*P *value	*P *trend^c^
	T1 (n = 29)	T2 (n = 25)	T3 (n = 27)		
BMI (kg/m²)^a^					
Model 1	23.4 ± 3.2	23.5 ± 3.5	23.5 ± 3.1	0.983	0.857
Model 2	23.4 ± 3.2	23.5 ± 3.2	23.4 ± 3.2	0.945	0.983
Waist circumference (cm)^a^					
Model 1	86.0 ± 10.2	84.8 ± 10.3	85.7 ± 9.8	0.725	0.828
Model 2	86.0 ± 6.5	84.7 ± 6.5	85.8 ± 6.5	0.422	0.848
Systolic blood pressure (mmhg)^a^					
Model 1	117.3 ± 12.6	110.9 ± 11.5	108.8 ± 14.0	0.308	0.135
Model 2	111.8 ± 11.3	110.8 ± 11.3	109.0 ± 11.4	0.301	0.126
Model 3^b^	112.6 ± 12.6	110.7 ± 11.2	108.4 ± 12.4	0.144	0.041
Diastolic blood pressure (mmhg)^a^					
Model 1	73.1 ± 6.0	74.0 ± 6.3	73.5 ± 6.7	0.611	0.626
Model 2	73.1 ± 6.5	73.9 ± 6.5	73.6 ± 6.5	0.735	0.633
Model 3^b^	74.0 ± 7.1	73.8 ± 6.3	72.9 ± 7.0	0.585	0.364
Fasting blood glucose (mg/dL)					
Model 1	175.1 ± 5.9	163.3 ± 5.5	143.0 ± 5.7	0.225	0.112
Model 2	168.7 ± 5.5	167.9 ± 5.5	145.2 ± 5.7	0.421	0.238
HbA1c^a^					
Model 1	7.0 ± 0.8	7.0 ± 0.8	7.0 ± 0.8	0.839	0.571
Model 2	7.0 ± 0.8	7.0 ± 0.8	7.0 ± 0.8	0.966	0.876
Total cholesterol (mg/dL)					
Model 1	171.7 ± 26.8	167.7 ± 37.0	169.8 ± 30.1	0.898	0.819
Model 2	170.1 ± 32.6	171.1 ± 33.4	168.3 ± 32.6	0.941	0.927
Model 3	174.0 ± 30.3	172.2 ± 31.1	163.1 ± 31.4	0.456	0.266
Triglyceride (mg/dL)					
Model 1	81.5 ± 1.6	63.5 ± 1.5	78.5 ± 1.4	0.075	0.703
Model 2	79.8 ± 5.8	61.9 ± 5.4	82.0 ± 5.6	0.038	0.856
Model 3	82.4 ± 5.8	63.5 ± 5.4	77.4 ± 5.7	0.079	0.487
HDL-C (mg/dL)					
Model 1	51.8 ± 9.8	55.1 ± 9.3	53.8 ± 9.4	0.429	0.425
Model 2	50.9 ± 9.4	55.5 ± 9.6	54.4 ± 9.3	0.223	0.232
Model 3	51.8 ± 9.9	55.9 ± 10.2	53.1 ± 10.3	0.203	0.551
LDL-C (mg/dL)					
Model 1	101.9 ± 5.7	92.3 ± 5.3	94.1 ± 5.5	0.344	0.327
Model 2	101.9 ± 5.7	95.9 ± 5.3	91.0 ± 5.5	0.329	0.134
Model 3	104.5 ± 5.7	95.1 ± 5.3	89.1 ± 5.5	0.130	0.033
LDL-C/HDL-C					
Model 1	2.1 ± 0.7	1.8 ± 0.7	1.8 ± 0.5	0.157	0.124
Model 2	2.1 ± 0.6	1.9 ± 0.6	1.7 ± 0.6	0.085	0.027
Model 3	2.1 ± 0.6	1.8 ± 0.6	1.8 ± 0.6	0.137	0.029
Superoxide dismutase activity (IU/mL)					
Model 1	35.2 ± 1.2	34.5 ± 1.1	34.6 ± 1.1	0.871	0.671
Model 2	35.4 ± 5.4	34.2 ± 5.3	34.5 ± 5.3	0.556	0.419
Glutathione peroxidase activity (IU/mL)					
Model 1	144.7 ± 3.4	125.2 ± 3.5	135.2 ± 3.4	0.911	0.835
Model 2	142.6 ± 6.7	126.2 ± 6.6	136.1 ± 6.5	0.906	0.902
Total antioxidant capacity (mmol/L)					
Model 1	362.5 ± 62.0	376.8 ± 65.9	379.9 ± 47.4	0.510	0.274
Model 2	357.6 ± 57.0	380.5 ± 59.1	381.5 ± 57.4	0.246	0.132

Data are shown as mean ± SD

Model 1 = crude, analysis of variance test was used. Model 2 = adjusted for age, sex, total energy intake (kcal/day), physical activity (MET/min/week), BMI (kg/m^2^), diabetes duration (year), total daily insulin dose (unit/day), education and dietary supplement intake. Model 3 = adjusted for age, sex, total energy intake (kcal/day), physical activity (MET/min/week), BMI (kg/m^2^), diabetes duration (year), total daily insulin dose (unit/day), education, dietary supplement intake, saturated fatty acid (g/day), mono-unsaturated fatty acid (g/day), poly-unsaturated fatty acid (g/day) and trans fatty acid (g/day), analysis of covariance test was used

*DPI* dietary phytochemical index, *BMI* body mass index, *HbA1c* hemoglobin A1c, *LDL-C* low-density lipoprotein cholesterol, *HDL-C* high-density lipoprotein cholesterol, *MET* metabolic equivalent

^a^T1 = 87, T2 = 87, T3 = 87. ^b^Adjusted for age, sex, total energy intake (kcal/day), physical activity (MET/min/week), BMI (kg/m^2^), diabetes duration (year), total daily insulin dose, education, dietary supplement intake, dietary intake of sodium (mg) and potassium (mg). ^c^*P *trend calculated by linear regression

**Fig. 3:**
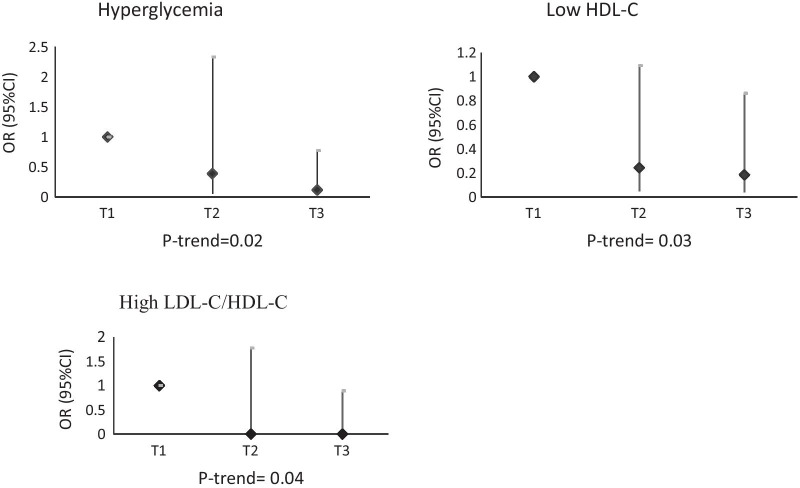
OR of cardiovascular risk factors in each tertile of dietary phytochemical index determined by multivariable logistic regression model with adjustment for age, sex, total energy intake (kcal/day), physical activity (metabolic equivalent task/hour/week), body mass index (kg/m^2^), diabetes duration (year), total insulin dose (unit/day), education and dietary supplement intake. *OR* odds ratio, *CI* confidence interval, *LDL-C* low-density lipoprotein cholesterol, *HDL-C* high-density lipoprotein cholesterol

The OR and 95% CI of CVD risk factors are shown in Table 3. In crude model, no significant association was found between CVD risk factors and DPI (*P *trend > 0.05), except for hyperglycemia that compared with participants in the lowest tertile, those in the highest tertile of DPI, had 85% lower chance of hyperglycemia (OR 0.15; 95% CI, 0.03–0.77). After adjustment for age, sex, total energy intake, physical activity, BMI, diabetes duration, total insulin dose, education and dietary supplement intake, compared with participants in the lowest tertile, those in the highest tertile of DPI, had 88% lower chance of hyperglycemia (OR 0.12; 95% CI, 0.02–0. 82), 81% lower chance of low HDL-C level (OR 0.19; 95% CI, 0.04–0.86) and 98% lower chance of high LDL-C/HDL-C ratio (OR 0.02; 95% CI, 0.001–0.89). In Fig. 3, the ORs of CVD risk factors across DPI tertiles are presented for statistically significant findings. No relationship was found between DPI and overweight or obesity, central obesity and HTN (*P *trend > 0.05). Although the chance of high LDL-C decreased across the DPI tertile in both crude and adjusted model, it was not significant (*P *trend > 0.05). All participants had DBP lower than 90 mmHg, so we were not able to calculate the OR for high DBP. Furthermore, just few participants (7 from 261) had hypertriglyceridemia, so logistic regression was not performed to assess the chance of hypertriglyceridemia. Because of the sparsity of data, wide 95% CI for high SBP was observed (Table 3).

**Table 3:** Odds ratio and 95% confidence intervals for cardiovascular risk factors across tertiles of sex‑specific energy‑adjusted DPI in patients with type 1 diabetes

Variable	Models	DPI tertile			*P *trend^b^
		T1 (n = 29)	T2 (n = 25)	T3 (n = 27)	
Hyperglycemia (> 100 mg/dL)	Model 1	1.0	0.38 (0.06–2.33)	0.15 (0.03–0.77)	0.016
	*P *value		0.326	0.022	
	Model 2	1.0	0.39 (0.05–3.14)	0.12 (0.02–0. 82)	0.020
	*P *value		0.485	0.031	
Low HDL-C (< 40 mg/dL in men and < 50 mg/dL in women)	Model 1	1.0	0.52 (0.16–1.69)	0.47 (0.14–1.52)	0.195
	*P *value		0.303	0.274	
	Model 2	1.0	0.24 (0.05–1.09)	0.19 (0.04–0.86)	0.033
	*P *value		0.064	0.039	
High LDL-C (> 100 mg/dL)	Model 1	1.0	0.60 (0.20–1.80)	0.54 (0.18–1.58)	0.254
	*P *value		0.487	0.333	
	Model 2	1.0	0.95 (0.26–3.46)	0.40 (0.11–1.44)	0.167
	*P *value		0.905	0.201	
Hypercholesterolemia (> 200 mg/dL)	Model 1	1.0	2.17 (0.46–10.16)	1.08 (0.20–5.89)	0.928
	*P *value		0.324	0.998	
	Model 2	1.0	4.59 (0.72–29.28)	1.20 (0.20–7.29)	0.882
	*P *value		0.114	0.832	
High LDL-C/HDL-C (> 3.5 in men, > 3 in women)	Model 1	1.0	0.33 (0.06–1.83)	0.48 (0.11–2.15)	0.299
	*P *value		0.222	0.302	
	Model 2	1.0	0.08 (0.004–1.77)	0.02 (0.001–0.89)	0.041
	*P *value		0.109	0.414	
High HbA1c (> 7%)^a^	Model 1	1.0	1.00 (0.55–1.81)	1.00 (0.55–1.81)	1.000
	*P *value		1.000	1.000	
	Model 2	1.0	1.10 (0.59–2.03)	1.12 (0.60–2.08)	0.722
	*P *value		0.898	0.729	
Overweight or obesity (BMI > 24.9 kg/m²)^a^	Model 1	1.0	1.06 (0.55–2.01)	0.79 (0.41–1.55)	0.498
	*P *value		0.973	0.554	
	Model 2	1.0	1.06 (0.54–2.08)	0.73 (0.37–1.48)	0.383
	*P *value		0.989	0.458	
Abdominal obesity (waist circumference > 94 cm in men and > 80 cm in women)^a^	Model 1	1.0	0.79 (0.43–1.44)	0.83 (0.45–1.51)	0.542
	*P *value		0.433	0.501	
	Model 2	1.0	0.49 (0.15–1.61)	0.47 (0.14–1.55)	0.201
	*P *value		0.210	0.244	
High systolic blood pressure (systolic blood pressure higher than 140 mmhg)^a^	Model 1	1.0	2.02 (0.18–22.73)	4.14 (0.45–37.85)	0.174
	*P *value		0.658	0.203	
	Model 2		1.59 (0.09–28.31)	4.15 (0.36–48.06)	0.205
	*P *value		0.887	0.342	

Logistic regression model was used

Model 1 = crude. Model 2 = adjusted for age, sex, total energy intake (kcal/day), physical activity (MET/min/week), BMI (kg/m^2^), diabetes duration (year), total insulin dose (unit/day), education and dietary supplement intake

*DPI* dietary phytochemical index, *LDL-C* low-density lipoprotein cholesterol, *HDL-C* high-density lipoprotein cholesterol, *HbA1c* hemoglobin A1c, *BMI* body mass index, *MET* metabolic equivalent

^a^T1 = 87, T2 = 87, T3 = 87

^b^*P *trend calculated by logistic regression test

## Discussion

In this cross sectional study, which investigated the relationship between DPI with CVD risk factors in patients with T1DM, we calculated the DPI based on the division of energy content of foods rich in phytochemicals. Our findings indicated that, participants with a higher DPI, had lower chance of high FBG, low HDL-C level, and high LDL-C to HDL-C ratio. Furthermore participants in the higher tertile of DPI had significantly lower LDL-C to HDL-C ratio, LDL-C and SBP compared with those in the lower tertile. Moreover, there were no relationships between DPI and other CVD risk factors.

In line with our findings, a longitudinal study reported significant inverse association between FBG level and DPI at baseline of study but not after 3-years of follow-up [23]. Furthermore, a case-control study has found that participants in the upper quartiles of DPI had lower odds of pre-diabetes [11]. Several studies reported significant association between intake of different phytochemicals and better FBG levels in healthy subjects [24, 25] and patients with type 2 DM [26, 27]. However, some studies have not found significant relationship between FBG level and DPI in healthy individuals [12, 28]. There are several mechanisms that phytochemicals influence carbohydrate metabolism and improve FBG [29], such as inhibition of carbohydrate digestion and intestinal glucose absorption, stimulation of insulin secretion from pancreatic β-cells, stimulation of hepatic glycolysis and glycogenesis, anti-inflammatory and antioxidant properties, effect on intracellular signaling pathway and gene expression [29, 30].

Our study revealed that higher intake of phytochemical rich food is associated with lower mean LDL-C and higher chance of high HDL-C levels. Golzarand et al. [31] observed that after 3 years of follow-up, the levels of TC, TG, HDL-C, and non-high-density lipoprotein cholesterol (non-HDL-C) in the highest quartile of DPI, significantly decreased in healthy men but not in women. Furthermore, our findings about HDL-C is in line with the study of Bahadoran et al. [12] that found higher mean serum HDL-C in the higher DPI tertile in healthy adults. However, in another study no association was reported between HDL-C level and DPI in healthy adults [28]. Different findings might be explained by the discrepancy in characteristics of participants, study design and study sample size.

Our study showed that higher intake of phytochemical rich food is significantly associated with lower chance of high LDL-C/HDL-C ratio, which is a predictor of CVD risk [32]. There are some studies that investigated the effect of phytochemicals intake on LDL-C/HDL-C ratio. A randomized clinical study on subjects with hypercholesterolemia, showed that intake of insoluble polyphenols could reduce the LDL-C/HDL-C ratio [33]. Another intervention study indicated that consumption of red wine polyphenols resulted in 7% reduction in LDL-C/HDL-C ratio compared to the baseline values in men who are at high risk of CVD [34]. Phytochemicals may improve lipid profile [35]. One of the best examples of a phytochemical-rich diet is Mediterranean diet. However, adding specific phytochemicals such as phytosterols and proanthocyanidins to Mediterranean diet increased the effect of diet on decreasing TC, LDL-C, and non-HDL-C and increasing HDL-C compared with Mediterranean diet alone [36]. Peroxisome proliferator-activated receptors (PPARs) are compounds that play an important role in lipid metabolism. Some phytochemicals as natural PPARs ligands could activate PPARs and affect lipid metabolism, increase fatty acid uptake, utilization, and catabolism by affecting on fatty acid transport. Furthermore, PPARs lead to lower plasma levels of cholesterol, triacylglycerol and higher HDL-C level [37].

Our findings showed that mean SBP decreased across increasing DPI tertiles, which is similar to the result of a study in healthy participants [12]. However, in logistic regression no association was found between SBP and DPI. On the contrary, some studies reported that participants with higher intake of phytochemical-rich diet had lower risk for occurrence of HTN in 3 years follow-up. However, no significant difference was observed in mean SBP and DBP across DPI quartiles [28]. Previous studies indicated that consumption of phytochemical rich food, such as fruits and vegetables [38], legumes [39] and olive [40], is inversely associated with HTN. Huang et al., reported that consumption of different sources of phytochemicals including soy isoflavones and berries polyphenols significantly reduced SBP but not DBP [41]. However, another study on participants with HTN observed that a combination of isolated phytochemicals and botanical extracts just decreased DBP but not SBP [42]. The dietary approaches to stop hypertension (DASH), which is rich in fruits, vegetables and low-fat dairy foods, reduces blood pressure. DASH diet provides an abundance of phytochemicals that may contribute to blood pressure lowering properties of this type of diet [43]. OS is an important factor in pathogenesis of HTN, that phytochemicals with antioxidant properties could reduce the risk of HTN. Flavonoids by modulating endothelial nitric oxide synthase activity, could increase endothelial-derived nitric oxide which is a vasodilator factor that enhances endothelial function [44].

In the present study, no association was found between antioxidant biomarkers and DPI. In line with our result, a previous cross-sectional study observed that DPI was not significantly related to total antioxidant statue in overweight adults [13]. In a cross-sectional study on healthy adolescents, higher intake of phytochemicals such as retinol, α-tocopherol, lycopene and the carotenoids was associated with higher serum levels of the mentioned antioxidant compounds, except lycopene [45]. The result of an experimental study showed no significant effect of phytosterol supplementation on plasma TAC in patients with metabolic syndrome [46]. Moreover, flavonoids supplement in patients with T1DM significantly decreased GPx activity, while other parameters of antioxidant capacity such as glutathione, activity of catalase and SOD remained unchanged [47]. These discrepancies could be explained by measuring different antioxidant biomarkers and various phytochemical supplementation.

This study has some limitations. The cross-sectional design is the major limitation of this study, which prevents to observe the cause and effect of the relationship. The other limitation is inherent limitations of DPI. In this index phytochemical rich food without energy content like spices, green and black tea were not considered. In addition, not considering the type of consumed phytochemicals is another limitation, because two diets with the same DPI but different source of phytochemicals could have different potential benefits. Although the various confounders were carefully controlled, it is possible to miss other residual confounders.

However, the strength of this study is investigating on dietary intake of patients with T1DM who are at high risk of several diabetic complications. Moreover, a validated and reliable FFQ was used which was completed by a trained dietitian.

## Conclusions

In conclusion, the findings of this study demonstrated that higher intake of phytochemical rich food, measured by DPI score was associated with lower chance of some CVD risk factors including dyslipidemia and high FBG in patient with T1DM. More studies more studies are warranted to corroborate the present findings.

## Data Availability

All data generated or analyzed during this study are included in this published article.

## Abbreviations

BMI: body mass index
CI: confidence interval
CVD: cardiovascular disease
DBP: diastolic blood pressure
DPI: dietary phytochemical index
DM: diabetes mellitus
FBG: fasting blood glucose
FCT: food composition tables
FFQ: food frequency questionnaire
GPx: glutathione peroxidase
HbA1c: hemoglobin A1c
HDL-C: high-density lipoprotein cholesterol
HTN: hypertension
IPAQ: international physical activity questionnaire
LDL-C: lipoprotein cholesterol
MET: metabolic equivalent task
MUFA: mono-unsaturated fatty acids
OR: odds ratio
OS: oxidative stress
PUFA: poly-unsaturated fatty acids
SBP: systolic blood pressure
SFA: saturated fatty acids
SOD: superoxide dismutase
T1DM: type1 diabetes mellitus
TAC: total antioxidant capacity
TG: triglyceride
TC: total cholesterol
USDA: US Department of Agriculture
WC: waist circumference

## References

[1] Brussels B: International Diabetes Federation.Diabetes and Cardiovascular Disease. In: International Diabetes Federation. 2016.

[2] Garralda-Del-Villar M, Carlos-Chillerón S, Diaz-Gutierrez J, Ruiz-Canela M, Gea A, Martínez-González MA, Bes-Rastrollo M, Ruiz-Estigarribia L, Kales SN, Fernández-Montero A (2019). Healthy lifestyle and incidence of metabolic syndrome in the SUN cohort. Nutrients.

[3] Rosen P, Nawroth PP, King G, Moller W, Tritschler HJ, Packer L (2001). The role of oxidative stress in the onset and progression of diabetes and its complications: a summary of a Congress Series sponsored by UNESCO-MCBN, the American Diabetes Association and the German Diabetes Society. Diabetes Metab Res Rev.

[4] Ayepola OR, Brooks NL, Oguntibeju OO (2014). Oxidative stress and diabetic complications: the role of antioxidant vitamins and flavonoids. Antioxidant-Antidiabetic Agents and Human Health.

[5] Varvařovská J, Racek J, Stožický F, Souček J, Trefil L, Pomahačová R (2003). Parameters of oxidative stress in children with type 1 diabetes mellitus and their relatives. J Diabetes Complicat.

[6] Dillard CJ, German JB (2000). Phytochemicals: nutraceuticals and human health. J Sci Food Agric.

[7] Firdous SM (2014). Phytochemicals for treatment of diabetes. EXCLI J.

[8] Han X, Shen T, Lou H (2007). Dietary polyphenols and their biological significance. Int J Mol Sci.

[9] McCarty MF (2004). Proposal for a dietary “phytochemical index”. Medical hypotheses.

[10] Bahadoran Z, Mirmiran P, Azizi F (2013). Dietary polyphenols as potential nutraceuticals in management of diabetes: a review. J Diabetes Metab Disorders.

[11] Abshirini M, Mahaki B, Bagheri F, Siassi F, Koohdani F, Sotoudeh G: Higher intake of phytochemical-rich foods is inversely related to prediabetes: A case-control study. Int J Prevent Med 2018, 9. 10.4103/ijpvm.IJPVM_145_18 PMC6085832 30147853

[12] Bahadoran Z, Golzarand M, Mirmiran P, Saadati N, Azizi F (2013). The association of dietary phytochemical index and cardiometabolic risk factors in adults: Tehran Lipid and Glucose Study. J Hum Nutr Dietetics.

[13] Vincent HK, Bourguignon CM, Taylor AG (2010). Relationship of the dietary phytochemical index to weight gain, oxidative stress and inflammation in overweight young adults. J Hum Nutr Dietetics.

[14] Vergès B (2009). Lipid disorders in type 1 diabetes. Diabetes Metab.

[15] Esfahani FH, Asghari G, Mirmiran P, Azizi F (2010). Reproducibility and relative validity of food group intake in a food frequency questionnaire developed for the Tehran Lipid and Glucose Study. J Epidemiol.

[16] Ghaffarpour M, Houshiar-Rad A, Kianfar H (1999). The manual for household measures, cooking yields factors and edible portion of foods. Tehran: Nashre Olume Keshavarzy.

[17] Azar M, Sarkisian E: Food composition table of Iran. Tehran: National Nutrition and Food Research Institute, Shaheed Beheshti University 1980, 65.

[18] Alberti KGMM, Zimmet P, Shaw J (2006). Metabolic syndrome—a new world-wide definition. A consensus statement from the international diabetes federation. Diabetic Med.

[19] Moghaddam MHB, Aghdam FB, Jafarabadi MA, Allahverdipour H, Nikookheslat SD, Safarpour S (2012). The Iranian Version of International Physical Activity Questionnaire (IPAQ) in Iran: content and construct validity, factor structure, internal consistency and stability. World Appl Sci.

[20] Stone NJ, Robinson JG, Lichtenstein AH, Merz CNB, Blum CB, Eckel RH, Goldberg AC, Gordon D, Levy D, Lloyd-Jones DM (2014). 2013 ACC/AHA guideline on the treatment of blood cholesterol to reduce atherosclerotic cardiovascular risk in adults: a report of the American College of Cardiology/American Heart Association Task Force on Practice Guidelines. J Am College Cardiol.

[21] Millán J, Pintó X, Muñoz A, Zúñiga M, Rubiés-Prat J, Pallardo LF, Masana L, Mangas A, Hernández-Mijares A, González-Santos P (2009). Lipoprotein ratios: physiological significance and clinical usefulness in cardiovascular prevention. Vasc Health Risk Manag.

[22] Willett W: Nutritional epidemiology. Oxford University Press, Oxford; 2012.

[23] Bahadoran Z, Mirmiran P, Tohidi M, Azizi F (2015). Dietary phytochemical index and the risk of insulin resistance and β-cell dysfunction: a prospective approach in Tehran lipid and glucose study. Int J Food Sci Nutr.

[24] Urquiaga I, D’Acuña S, Pérez D, Dicenta S, Echeverría G, Rigotti A, Leighton F (2015). Wine grape pomace flour improves blood pressure, fasting glucose and protein damage in humans: a randomized controlled trial. Biol Res.

[25] Almoosawi S, Fyfe L, Ho C, Al-Dujaili E (2010). The effect of polyphenol-rich dark chocolate on fasting capillary whole blood glucose, total cholesterol, blood pressure and glucocorticoids in healthy overweight and obese subjects. Br J Nutr.

[26] Lu T, Sheng H, Wu J, Cheng Y, Zhu J, Chen Y (2012). Cinnamon extract improves fasting blood glucose and glycosylated hemoglobin level in Chinese patients with type 2 diabetes. Nutr Res.

[27] Rahimi HR, Mohammadpour AH, Dastani M, Jaafari MR, Abnous K, Mobarhan MG, Oskuee RK (2016). The effect of nano-curcumin on HbA1c, fasting blood glucose, and lipid profile in diabetic subjects: a randomized clinical trial. Avicenna J Phytomed.

[28] Bahadoran Z, Golzarand M, Mirmiran P, Amouzgar A, Azizi F (2012). Association between dietary phytochemical index and occurrence of metabolic syndrome and its risk factors (among Tehranian adults): Tehran Lipid and Glucose Study. Iran J Endocrinol Metab.

[29] Hanhineva K, Törrönen R, Bondia-Pons I, Pekkinen J, Kolehmainen M, Mykkänen H, Poutanen K (2010). Impact of dietary polyphenols on carbohydrate metabolism. Int J Mol Sci.

[30] Vinayagam R, Xiao J, Xu B (2017). An insight into anti-diabetic properties of dietary phytochemicals. Phytochem Rev.

[31] Golzarand M, Mirmiran P, Bahadoran Z, Alamdari S, Azizi F (2014). Dietary phytochemical index and subsequent changes of lipid profile: A 3-year follow-up in Tehran Lipid and Glucose Study in Iran. ARYA atheroscler.

[32] Buchwald H, Boen JR, Nguyen PA, Williams SE, Matts JP (2001). Plasma lipids and cardiovascular risk: a POSCH report. Program on the Surgical Control of the Hyperlipidemias. Atherosclerosis.

[33] Ruiz-Roso B, Quintela JC, de la Fuente E, Haya J, Perez-Olleros L (2010). Insoluble carob fiber rich in polyphenols lowers total and LDL cholesterol in hypercholesterolemic sujects. Plant Foods Hum Nutr.

[34] Chiva-Blanch G, Urpi-Sarda M, Ros E, Valderas-Martinez P, Casas R, Arranz S, Guillen M, Lamuela-Raventos RM, Llorach R, Andres-Lacueva C (2013). Effects of red wine polyphenols and alcohol on glucose metabolism and the lipid profile: a randomized clinical trial. Clin Nutr.

[35] Cicero AF, Colletti A (2016). Role of phytochemicals in the management of metabolic syndrome. Phytomedicine.

[36] Jones JL, Fernandez ML, McIntosh MS, Najm W, Calle MC, Kalynych C, Vukich C, Barona J, Ackermann D, Kim JE (2011). A Mediterranean-style low-glycemic-load diet improves variables of metabolic syndrome in women, and addition of a phytochemical-rich medical food enhances benefits on lipoprotein metabolism. J Clin Lipidol.

[37] Ehrmann J, Vavrusova N, Collan Y, Kolar Z (2002). Peroxisome proliferator-activated receptors (PPARs) in health and disease. Biomed Pap Palacky UniversIN OLOMOUC.

[38] Wu L, Sun D, He Y (2016). Fruit and vegetables consumption and incident hypertension: dose–response meta-analysis of prospective cohort studies. J Hum Hypertens.

[39] Jayalath VH, De Souza RJ, Sievenpiper JL, Ha V, Chiavaroli L, Mirrahimi A, Di Buono M, Bernstein AM, Leiter LA, Kris-Etherton PM (2013). Effect of dietary pulses on blood pressure: a systematic review and meta-analysis of controlled feeding trials. Am J Hypertens.

[40] Martín-Peláez S, Castañer O, Konstantinidou V, Subirana I, Muñoz-Aguayo D, Blanchart G, Gaixas S, de la Torre R, Farré M, Sáez GT (2017). Effect of olive oil phenolic compounds on the expression of blood pressure-related genes in healthy individuals. Eur J Nutr.

[41] Huang H, Chen G, Liao D, Zhu Y, Xue X (2016). Effects of berries consumption on cardiovascular risk factors: a meta-analysis with trial sequential analysis of randomized controlled trials. Sci Rep.

[42] Biesinger S, Michaels H, Quadros A, Qian Y, Rabovsky A, Badger R, Jalili T (2016). A combination of isolated phytochemicals and botanical extracts lowers diastolic blood pressure in a randomized controlled trial of hypertensive subjects. Eur J Clin Nutr.

[43] Most MM (2004). Estimated phytochemical content of the dietary approaches to stop hypertension (DASH) diet is higher than in the Control Study Diet. J Am Dietetic Assoc.

[44] Cassidy A, O’Reilly ÉJ, Kay C, Sampson L, Franz M, Forman J, Curhan G, Rimm EB (2010). Habitual intake of flavonoid subclasses and incident hypertension in adults. Am J Clin Nutr.

[45] Neuhouser ML, Rock CL, Eldridge AL, Kristal AR, Patterson RE, Cooper DA, Neumark-Sztainer D, Cheskin LJ, Thornquist MD (2001). Serum concentrations of retinol, α-tocopherol and the carotenoids are influenced by diet, race and obesity in a sample of healthy adolescents. J Nutr.

[46] Sialvera TE, Koutelidakis AE, Richter DJ, Yfanti G, Kapsokefalou M, Micha R, Goumas G, Diamantopoulos E, Zampelas A (2013). Phytosterol supplementation does not affect plasma antioxidant capacity in patients with metabolic syndrome. Int J Food Sci Nutr.

[47] Manuel y Keenoy B, Vertommen J, De Leeuw I (1999). The effect of flavonoid treatment on the glycation and antioxidant status in Type 1 diabetic patients. Diabetes Nutr Metab.

